# Pretargeted Imaging with Gallium-68—Improving the Binding Capability by Increasing the Number of Tetrazine Motifs

**DOI:** 10.3390/ph11040102

**Published:** 2018-10-11

**Authors:** Dominik Summer, Sonja Mayr, Milos Petrik, Christine Rangger, Katia Schoeler, Lisa Vieider, Barbara Matuszczak, Clemens Decristoforo

**Affiliations:** 1 Department of Nuclear Medicine, Medical University Innsbruck, Anichstrasse 35, A-6020 Innsbruck, Austria; dominik.summer@i-med.ac.at (D.S.); sonja.mayr@student.uibk.ac.at (S.M.); christine.rangger@i-med.ac.at (C.R.); 2 Institute of Molecular and Translational Medicine, Faculty of Medicine and Dentistry, Palacky University, CZE-77900 Olomouc, Czech Republic; milos.petrik@seznam.cz; 3 Biocenter—Division of Developmental Immunology, Medical University of Innsbruck, Innrain 80/82, A-6020 Innsbruck, Austria; katia.schoeler@i-med.ac.at; 4 Institute of Pharmacy, Pharmaceutical Chemistry, University of Innsbruck, Center for Chemistry and Biomedicine (CCB), Innrain 80/82, A-6020 Innsbruck, Austria; lisa.vieider@student.uibk.ac.at (L.V.); barbara.matuszczak@uibk.ac.at (B.M.)

**Keywords:** pretargeting, Fusarinine C, rituximab, click chemistry, multimerization, PET, gallium-68

## Abstract

The inverse electron-demand Diels-Alder reaction between 1,2,4,5-tetrazine (Tz) and *trans*-cyclooct-2-ene (TCO) has gained increasing attraction among extensive studies on click chemistry due to its exceptionally fast reaction kinetics and high selectivity for in vivo pretargeting applications including PET imaging. The facile two-step approach utilizing TCO-modified antibodies as targeting structures has not made it into clinics yet. An increase in the blood volume of humans in comparison to mice seems to be the major limitation. This study aims to show if the design of multimeric Tz-ligands by chelator scaffolding can improve the binding capacity and may lead to enhanced PET imaging with gallium-68. We utilized for this purpose the macrocyclic siderophore Fusarinine C (FSC) which allows conjugation of up to three Tz-residues due to three primary amines available for site specific modification. The resulting mono- di- and trimeric conjugates were radiolabelled with gallium-68 and characterized in vitro (logD, protein binding, stability, binding towards TCO modified rituximab (RTX)) and in vivo (biodistribution- and imaging studies in normal BALB/c mice using a simplified RTX-TCO tumour surrogate). The ^68^Ga-labelled FSC-based Tz-ligands showed suitable hydrophilicity, high stability and high targeting specificity. The binding capacity to RTX-TCO was increased according to the grade of multimerization. Corresponding in vivo studies showed a multimerization typical profile but generally suitable pharmacokinetics with low accumulation in non-targeted tissue. Imaging studies in RTX-TCO tumour surrogate bearing BALB/c mice confirmed this trend and revealed improved targeting by multimerization as increased accumulation in RTX-TCO positive tissue was observed.

## 1. Introduction

Immunoglobulins, in particular monoclonal antibodies (mAbs) are highly attractive targeting structures due to their extraordinary specificity and selectivity and are well established for therapeutic applications, particularly in the field of oncology [1,2]. Because of their favorable targeting abilities and therefore an ever increasing clinical importance, mAbs have regained interest also as imaging agents, in particular for positron emission tomography (PET) applications [3,4,5]. In principle mAbs can be directly radiolabelled either by direct incorporation of radiohalogens or by attaching a chelator to the protein in case of radiometals. Prolonged circulation time and slow distribution within the organism, however, restricts its use to long-lived radionuclides e.g., zirconium-89 (3.26 d) and iodine-124 (4.18 d). This multiday circulation paired with slow radioactive decay provides unfavorable radiation to healthy tissue and adds significantly to the overall radiation burden of patients.

In order to overcome this problem, various pretargeting methodologies have been reported and reviewed recently [6,7,8], enabling a straight forward two-step approach. Thereby the modified antibody is administered, allowed to accumulate at the target site and be eliminated from the bloodstream followed by injection of the radioactive payload to form the radioimmunoconjugate in vivo. This provides certain advantages as it facilitates the use of short-lived radioisotopes for PET applications e.g., gallium-68 (1.13 h), fluorine-18 (1.83 h) and copper-64 (12.7 h) and significantly reduces the radiation dose to healthy tissue, since the radioligand either finds its binding partner to form stable conjugates or is rapidly eliminated due to its small size. Furthermore, it allows to apply radiolabelling at high temperatures using high concentrations of organic solvents if necessary—harsh conditions, where the structural integrity of antibodies would be severely in danger when direct labelling was performed.

Among these pretargeting strategies the inverse electron-demand Diels-Alder reaction (IEDDA) between 1,2,4,5-tetrazines (Tz) and *trans*-cyclooct-2-enes (TCO) has gained enormous attention mainly due to the exceptionally fast reaction kinetics and high selectivity between the reaction partners even in complex biological systems as encountered in vivo [9,10]*.* Various preclinical studies demonstrated the feasibility of this approach for molecular imaging using PET-radioisotopes with promising results [11,12,13,14]. The application on humans, however, remains unsuccessful due to the increased blood volume in humans and may therefore lead to insufficient accumulation due to accelerated elimination of the small-sized radioligand.

**Scheme 1:**
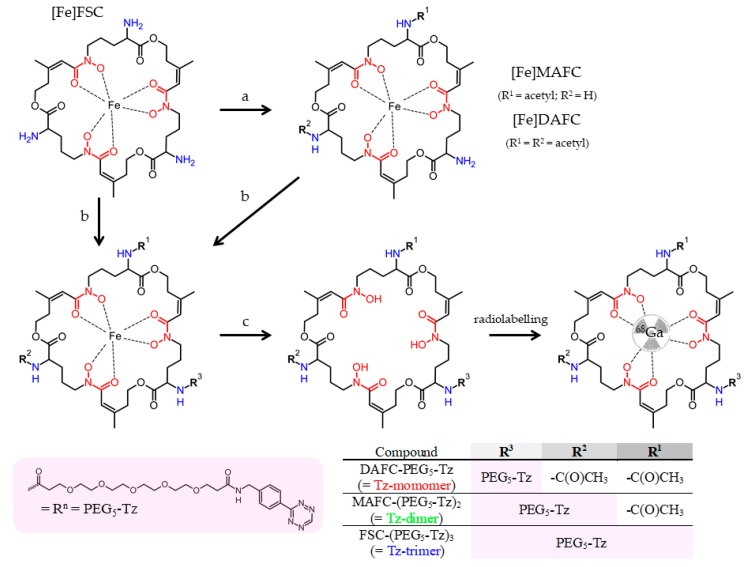
Synthetic strategy for FSC-based tetrazine (Tz) conjugates [a: methanol and acetic anhydride (MetOH/Ac_2_O); b: Tz-PEG_5_-NHS/DMF/DIPEA; c: EDTA] radiolabelled with gallium-68.

Related to this, recent study investigated whether an increase of Tz motifs by chelator scaffolding, i.e., multimerization, can improve the binding efficiency and thereby improve imaging contrast. This could contribute in particular to the application of gallium-68 for pretargeted immuno-PET imaging. For this purpose we utilized the macrocyclic chelator Fusarinine C for the design of mono- and multimeric Tz-conjugates as presented in Scheme 1 for a proof-of-concept study, on potentially improved pretargeting for imaging with gallium-68 by applying multimerization. We chose non-internalizing anti-CD20 antibody rituximab (RTX) modified with TCO as targeting vector. Radiolabelling was conducted at room temperature within minutes and the ^68^Ga-labelled conjugates showed reasonable hydrophilicity and excellent stability in human serum. Protein binding, however, remained comparable within the conjugates but was generally high. The ^68^Ga-labelled multimeric conjugates showed a higher binding capacity towards TCO-motif bearing RTX. Furthermore, cell-binding studies revealed highly specific targeting properties and the binding of [^68^Ga]Ga-FSC-Tz multimers to CD20-expressing Raji cells increased with the number of Tz-motifs attached to the chelator. Imaging studies in a simplified pretargeting mouse model proved the trend for improved targeting. We therefore conclude, that multimerization bears a great potential to improve IEDDA related pretargeting when short-lived PET-radioisotopes, particularly gallium-68, are used. Further investigations in established tumor models are warranted to confirm these promising findings.

## 2. Results

### 2.1. (Radio) Chemistry

FSC-based mono- and multimeric Tz-conjugates were accessible in a three-step synthesis to give the corresponding conjugates in good yields and high chemical purity (>95%; analytical RP-HPLC, UV absorption at λ = 220 nm). The results from mass analysis were in good agreement with the calculated values. The structure of the Tz-conjugates was further confirmed by ^1^H-NMR spectroscopy. The singlets at 6.30 and 1.86 ppm which can be assigned to the -C=O-CH=C(CH_3_)C-substructure were used as the marker signals of the FSC subunit. The singlet at 1.83 ppm corresponds to the methyl protons of the acetyl group(s). The singlet at 10.56 ppm is highly characteristic for the tetrazine moiety. The doublets at 8.44 and 7.53 ppm with coupling constants of 8.4 Hz are characteristic for the para-substituted phenyl ring, and the triplet at 8.50 ppm as well as the doublet at 4.40 ppm with a coupling constant of 6.0 Hz can be assigned to the -NH-CH_2_ group of the PEG_5_-Tz subunit(s). The ratio of the integrals of these marker signals is summarized in Table 1 and corresponding ^1^H-NMR spectra are presented in Figures S1–S3. Radiolabelling with gallium-68 was quantitative within minutes at RT, thus exhibiting fast labelling kinetics. Corresponding (radio-)RP-HPLC chromatograms are presented in Figure S4.

### 2.2. In Vitro Evaluation

Stability studies of ^68^Ga-labelled conjugates in fresh human serum and PBS as control revealed high stability as no major degradation was observed over a period of 4 h. Corresponding radio-RP-HPLC chromatograms are presented in Figure S5. The results of logD studies and the ability to bind to serum proteins of ^68^Ga-labelled conjugates are summarized in Table 2. They revealed suitable hydrophilicity with minor decrease when increasing the number of Tz residues. All conjugates showed very high protein binding with minor differences between mono- and multimeric [^68^Ga]Ga-Tz-ligands.

The non-internalizing anti-CD20 monoclonal antibody rituximab was modified with the TCO motif similar to a previously published procedure [15] and corresponding FACS analysis of CD20-expressing Raji cells incubated with both, TCO-modified and non-modified RTX showed high target specificity (Figure S6), thus demonstrating that the binding ability was not altered by the TCO modification.

**Figure 1:**
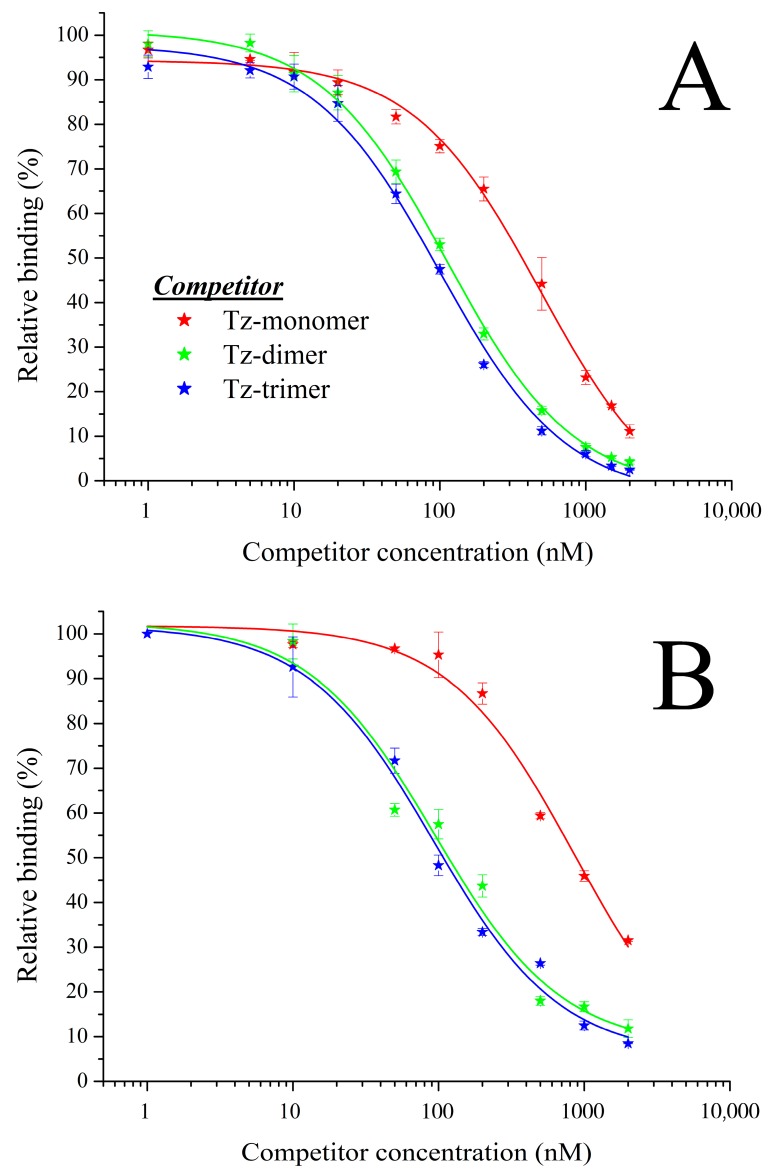

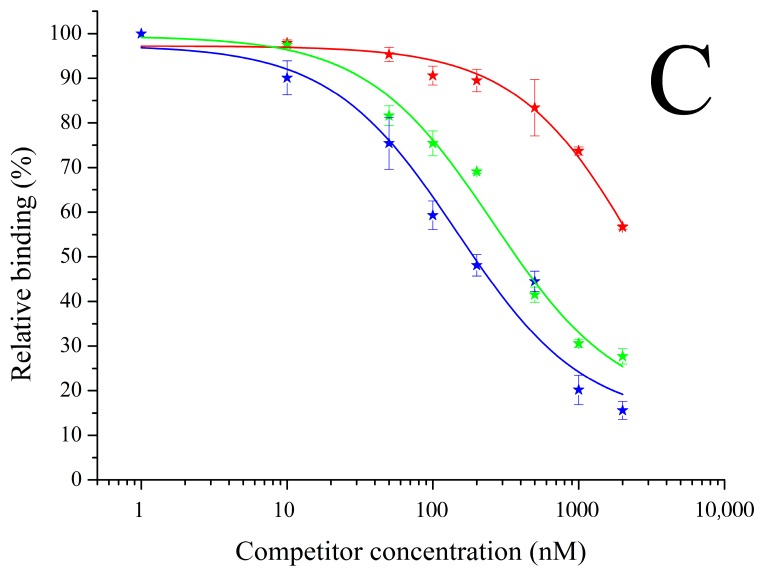
Competitive binding studies of [^68^Ga]Ga-Tz-monomer (**A**), [^68^Ga]Ga-Tz-dimer (**B**) and [^68^Ga]Ga-Tz-trimer (**C**) on immobilized RTX-TCO using the non-labelled counterparts as competitor.

The binding capacity of ^68^Ga-labelled mono- and multimeric Tz-ligands was assessed via competitive binding on immobilized RTX-TCO using the non-labelled conjugates as competitor and is presented in Figure 1. The binding of the [^68^Ga]Ga-Tz-monomer was reduced by 50% at a competitor concentration of 486 ± 52 nM when challenged with the non-labelled monomer, whereas the non-labelled dimer (112 ± 6 nM) and trimer (100 ± 10 nM) reduced the binding at significantly lower concentrations. The binding of the [^68^Ga]Ga-Tz-dimer was reduced by half at 95 ± 25 nM in competition with its non-labelled counterpart and at a comparable concentration with the non-labelled trimer (92 ± 15 nM), whereas a decrease to 50% was only achieved at a much higher concentration in competition with non-labelled monomer (865 ± 263 nM). Binding studies of the [^68^Ga]Ga-Tz-trimer showed a comparable trend as the non-labelled trimer reduced the binding by 50% at 147 ± 49 nM and the dimer at 258 ± 60 nM; whereby a significantly higher amount of the monomer (2987 ± 1664 nM) was needed for a 50% binding reduction. Overall in all assays improved binding of di- and trimer over monomer was observed.

**Figure 2:**
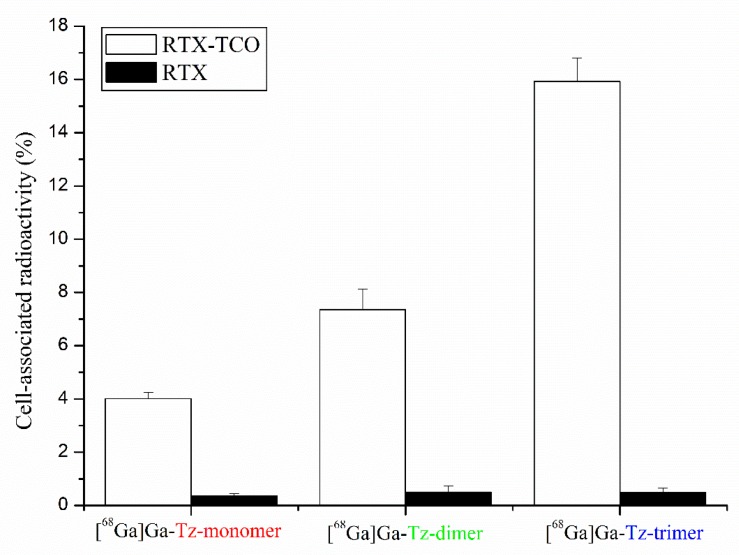
Cell-binding studies of ^68^Ga-labelled FSC-based Tz-ligands on CD20-expressing Raji cells pre-treated with anti-CD20 antibody RTX (negative control, black bars) and its TCO modified counterpart (white bars).

The results of cell-binding studies on CD20-expressing Raji cells pre-treated with RTX or RTX-TCO prior to incubation with ^68^Ga-labelled Tz-ligands are presented in Figure 2. All ^68^Ga-labelled conjugates showed highly specific targeting properties as the amount of unspecific bound radioligand to RTX pre-treated Raji cells was negligible low (<1%). The binding of ^68^Ga-labelled Tz-ligands on RTX-TCO bound Raji cells increased with the grade of multimerization and was 4.01 ± 0.24% for [^68^Ga]Ga-Tz-monomer, 7.35 ± 0.77% for [^68^Ga]Ga-Tz-dimer and 15.93 ± 0.88 for [^68^Ga]Ga-Tz-trimer, respectively.

### 2.3. In Vivo Evaluation

**Figure 3:**
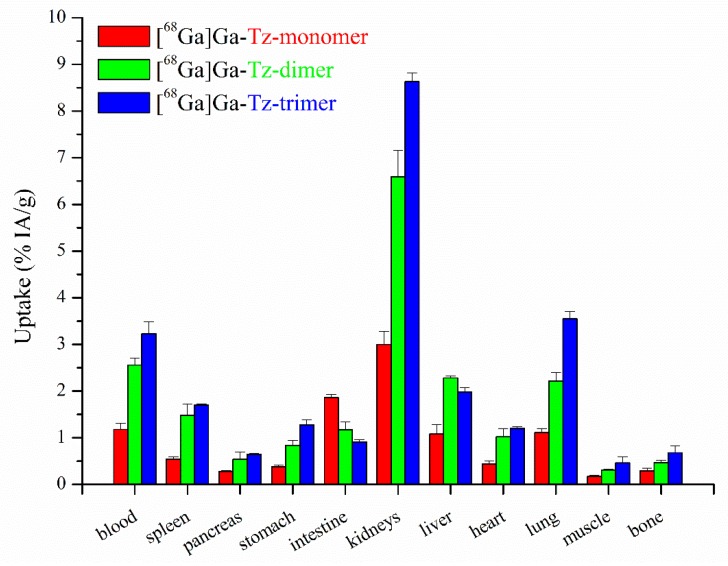
Biodistribution studies of ^68^Ga-labelled mono- and multimeric FSC-based pretargeting agents in normal BALB/c mice 1 h p.i. presented as percentage of total injected activity per gram tissue (*n* = 3).

Biodistribution studies in non-tumour xenografted BALB/c mice 1 h after administration of the ^68^Ga-labelled Tz-ligands are shown in Figure 3. In general, accumulation in non-targeted tissue was significantly lower for the [^68^Ga]Ga-Tz-monomer compared to the [^68^Ga]Ga-Tz-trimer, whereby the difference between the multimers was less pronounced. In particular, the multimeric Tz-ligands showed slower blood clearance and higher accumulation in renal tissue compared to the monomeric conjugate. The conjugates generally showed low accumulation in non-targeted tissue and low retention in critical organs (e.g., muscle, bone).

**Figure 4:**
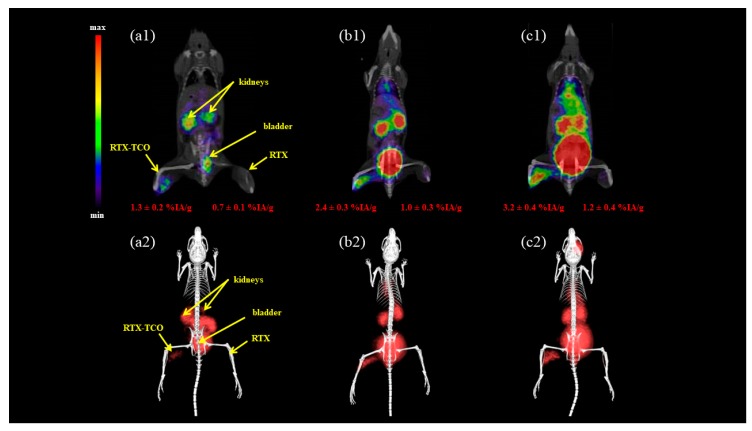
Static µPET/CT image of i.m. RTX(-TCO) pre-treated normal BALB/c mice 5 h after treatment and 90 min p.i. of the ^68^Ga-labelled Tz-monomer (**a**), Tz-dimer (**b**) and Tz-trimer (**c**). (1 = coronal slice; 2 = 3D volume rendered projections; both prone position).

Imaging studies using BALB/c mice i.m. injected with RTX and RTX-TCO as tumour surrogate 5 h prior to r.o. administration of the radioligand and imaging performed 90 min p.i. are presented in Figure 4. The results mainly confirmed the suitable in vivo distribution profile of FSC-based Tz-ligands radiolabelled with gallium-68 with main activity in kidneys and bladder and some blood pool activity for the ^68^Ga-labelled multimers. Moreover, the accumulation in RTX-TCO positive muscle tissue increased with ascending numbers of Tz motifs and was ~1% for the [^68^Ga]Ga-Tz-monomer, ~2% for the [^68^Ga]Ga-Tz-dimer and ~3% for the [^68^Ga]Ga-Tz-trimer. Surprisingly, the accumulation in TCO negative tissue was also approximately 1% showing negligible differences between the different radioligands.

## 3. Discussion

IEDDA-based pretargeting has become increasingly popular for molecular imaging as well as radioimmunotherapy over the past few years [16,17,18,19,20,21]. Despite recent advancements towards structural improvement of cyclen- and TACN-based Tz-probes [22,23] or the synthesis of TCO-bearing dendrimers [24], the design of targeting probes bearing multiple Tz-motifs for potentially improved pretargeting has scarcely been investigated. Devaraj and co-workers reported on polymer modified tetrazines (PMT) radiolabelled with fluorine-18 [25] and gallium-68 [26] for PET applications while Zlatni et al. recently demonstrated the targeting applicability for microbubbles bearing multiple Tz-motifs towards prostate specific membrane antigen for ultrasound related diagnostic purposes [15]. However, investigations on the use of small-sized multimeric Tz-ligands have not been reported yet.

FSC is a suitable chelating scaffold for PET radiometals, particularly gallium-68 and zirconium-89 [27,28]. Its unique structural properties enable straight forward mono- and multimeric tracer design [29,30,31,32]. This novel chelator, therefore, represented a very suitable scaffold for the synthesis of mono- and multimeric small-sized Tz-bearing probes for radiolabelling with gallium-68 to evaluate potential benefits for IEDDA-based pretargeting by increasing the number of Tz-residues. The resulting conjugates showed reasonable hydrophilicity and increasing the number of Tz-residues from one to three did not alter the water solubility too much, although being somewhat lower in comparison to linker modified monomeric conjugates [23]. Unexpectedly, all FSC-based Tz-conjugates showed high protein binding with negligible differences between the compounds. This can be related to the introduction of the pegylated Tz residue, since non-modified ^68^Ga-labelled FSC-based precursors showed a protein binding of <3% (Kaepookum et al. unpublished results). This phenomenon has not been reported in the case of monomeric DOTA- and TACN-based derivatives [22,23] and might be disadvantageous by slowing down the distribution of the tracer to the TCO-interaction site. In vivo results, however, did not really reveal a major problem with too slow pharmacokinetics, since the washout from non-targeted tissue was still sufficiently rapid. Competitive binding assessments showed significantly reduced binding of [^68^Ga]Ga-Tz-monomer by a factor of five when challenged with the multimeric conjugates in comparison with the non-labelled monomer. This corresponded with the significantly higher amounts of Tz-monomer needed, 8 to 20 fold respectively, to reduce the binding of the ^68^Ga-labelled multimers by 50%, thus indicating that the binding capacity increased with the number of Tz residues. This was substantiated by the results of the cell binding studies, since the binding to CD20-expressing Raji cells increased by a factor of 1.8 for the [^68^Ga]Ga-Tz-dimer and 3.9 for the [^68^Ga]Ga-Tz-trimer in comparison to the [^68^Ga]Ga-Tz-monomer. Biodistribution studies in non-tumour xenografted healthy BALB/c mice indicated suitable pharmacokinetics and showed a multimerization typical profile exhibiting slower blood clearance and increased kidney retention, similar to prior findings with the FSC-scaffold [31,32]. Imaging studies using a tumour surrogate model confirmed this trend and demonstrated increased binding of the ^68^Ga-labelled multimeric Tz-conjugates. The accumulation in RTX-TCO pretreated muscle tissue doubled for the [^68^Ga]Ga-Tz-dimer and was 3-fold higher for the [^68^Ga]Ga-Tz-trimer compared to the [^68^Ga]Ga-Tz-monomer.

In regard to imaging in living mice there was a clear improvement of target specific accumulation when switching from mono- to multimers. The use of our simplified tumour surrogate animal model, however, requires further investigations in established animal models to address the limitations of this study:

(1) The accumulation in non-targeted tissue, i.e., i.m. injection of RTX resulted to be ~1%. This effect was even more pronounced when choosing a shorter time interval of 2 h between i.m. administration of the mAb and radioligand injection (data not shown) and was only seen at the injection site but generally not in other tissue. We speculate that this is related to the injection of the antibody leading to tissue damage with increased tissue permeability and unspecific accumulation of the radioligand.

(2) Intravenously injected mAb stimulates accumulation at the target interaction site, but a non-negligible amount remains in circulation, thereby increasing the background signal when administering the radioligand. In order to reduce this amount, high molecular weight TCO-scavenger molecules exhibiting low vascular permeability (clearing agents) have been established with great success, significantly improving target-to-background (TTB) ratios [33,34]. The need for clearing agents was completely neglected in this study and we are fully aware that our animal model does not reflect the real situation regarding TTB ratios.

In summary, we have been able to show that FSC is a suitable scaffold for the design of multimeric Tz-conjugates for radiolabelling with gallium-68. Although multimeric Tz-ligands exhibited significant improvements towards IEDDA-based pretargeting additional studies in tumour models are warranted to explore the full potential of this promising concept. Furthermore, a highly interesting therapeutic approach is the release of drugs directly at the interaction site from antibody-drug conjugates (ADCs) mediated via IEDDA reaction between Tz and TCO [35,36,37]. It might be of interest for future perspectives if multimeric Tz-conjugates can boost the release of drugs improving this highly promising “click-to-release” strategy.

## 4. Materials and Methods

### 4.1. Instrumentation

#### 4.1.1. Analytical [radio]-RP-HPLC

Reversed-phase high-performance liquid chromatography analysis was performed with the following instrumentation: UltiMate 3000 RS UHPLC pump, UltiMate 3000 autosampler, UltiMate 3000 column compartment (25 °C oven temperature), UltiMate 3000 Variable Wavelength Detector (Dionex, Germering, Germany; UV detection at λ = 220 nm) a radio detector (GabiStar, Raytest; Straubenhardt, Germany), Jupiter 5 μm C_18_ 300 Å 150 × 4.6 mm (Phenomenex Ltd. Aschaffenburg, Germany) column with acetonitrile (ACN)/H_2_O/0.1% trifluoroacetic acid (TFA) as mobile phase; flow rate of 1 mL/min; gradient: 0.0–1.0 min 10% ACN, 1.0–12.0 min 10–60% ACN, 13.0–15.0 min 60–80% ACN, 15.0–16.0 min 80–10% ACN, 16.0–20.0 min 10% ACN.

#### 4.1.2. Preparative RP-HPLC

Sample purification via RP-HPLC was carried out as follows: Gilson 322 Pump with a Gilson UV/VIS-155 detector (UV detection at λ = 220 nm) using a PrepFC™ automatic fraction collector (Gilson, Middleton, WI, USA), Eurosil Bioselect Vertex Plus 30 × 8 mm 5 μm C_18A_ 300 Å pre-column and Eurosil Bioselect Vertex Plus 300 × 8 mm 5 μm C_18A_ 300 Å column (Knauer, Berlin, Germany) and following ACN/H_2_O/0.1% TFA gradients with a flow rate of 2 mL/min: gradient A: 0.0–5.0 min 0% ACN, 5.0–35.0 min 0–50% ACN, 35.0–38.0 min 50% ACN, 38.0–40.0 min 50–0% ACN. Gradient B: 0.0–5.0 min 10% ACN, 5.0–40.0 min 10–60% ACN, 41.0–45.0 min 60% ACN, 46.0–50.0 min. 60–80% ACN, 51.0–55.0 min 80–10% ACN.

#### 4.1.3. MALDI-TOF MS

Mass spectrometry was conducted on a Bruker microflex^TM^ bench-top MALDI-TOF MS (Bruker Daltonics, Bremen, Germany) with a 20 Hz laser source. Sample preparation was performed according to dried-droplet method on a micro scout target (MSP96 target ground steel BC, Bruker Daltonics) using α-cyano-4-hydroxycinnamic acid (HCCA, Sigma-Aldrich, Handels GmbH, Vienna, Austria) as matrix. Flex Analysis 2.4 software was used for processing of the recorded data.

#### 4.1.4. ^1^H-NMR Spectroscopy

^1^H-NMR spectra of the FSC-based Tz-conjugates were recorded on a “Saturn” 600 MHz Avance II+ spectrometer and the NMR of the reference compound (TAFC) was recorded on a “Mars” 400 MHz Avance 4 Neo spectrometer, both from Bruker Corporation (Billerica, MA, USA). The centre of the solvent multiplet (DMSO-*d*_6_) was used as internal standard (chemical shifts in δ ppm), which was related to TMS with δ 2.49 ppm. TopSpin 3.5 pl7 software (Bruker) was used for data processing.

### 4.2. Synthesis

#### 4.2.1. General Information

All chemicals and solvents were purchased as reagent grade from commercial sources unless otherwise stated. *trans*-Cyclooctene-NHS ester and tetrazine-PEG_5_-NHS ester were bought from Click Chemistry Tools (Scottsdale, AZ, USA). Rituximab (MabThera^®^, Roche Pharma AG, Grenzach-Wyhlen, Germany) was of pharmaceutical grade and was a kind gift from the University Hospital of Innsbruck.

#### 4.2.2. [Fe]Fusarinine C ([Fe]FSC)

The cyclic siderophore Fusarinine C (FSC) was obtained from iron deficient fungal culture and the extraction of FSC was conducted with a slightly modified method as described before [38]. Briefly, 1 L of iron saturated culture media was flushed through a C_18_-Reveleris flash cartridge (40 μm, 12 g; Grace, MD, USA) by using a REGLO tubing pump (Type ISM795, Ismatec SA, Glattbrugg-Zurich, Switzerland) with a flow rate of 10 mL/min. [Fe]FSC fixed on the cartridge was washed with 50 mL of water and eluted afterwards with 50 mL H_2_O/ACN (20/80% *v*/*v*). After evaporation to dryness ~300 mg [Fe]FSC were obtained as red−brown coloured solid in high purity (>90%). Analytical data: RP-HPLC *t*_R_ = 6.95 min; MALDI TOF-MS: *m*/*z* [M + H]^+^ = 779.93 [C_33_H_51_FeN_6_O_12_; exact mass: 779.63 (calculated)].

#### 4.2.3. Acetylation of [Fe]FSC

[Fe]FSC was dissolved in methanol to a final concentration of 30 mg/mL (38.5 mM). An aliquot of 300 µL was reacted with 10 µL of acetic anhydride for 5 min at room temperature (RT) under vigorous shaking followed by subsequent purification of the resulting mixture of mono-, di- and triacetylfusarinine C via preparative RP-HPLC using gradient A to obtain *N*-monoacetylfusarinine C ([Fe]MAFC, *t*_R_ = 20.1 min) and *N*,*N’*-diacetylfusarinine C ([Fe]DAFC, *t*_R_ = 24.5 min). Analytical data: [Fe]MAFC: RP-HPLC *t*_R_ = 7.67 min; MALDI TOF-MS: *m*/*z* [M + H]^+^ = 822.04 [C_35_H_53_FeN_6_O_13_; exact mass: 821.67 (calculated)]. [Fe]DAFC: RP-HPLC *t*_R_ = 8.49 min; MALDI TOF-MS: *m*/*z* [M + H]^+^ = 864.02 [C_37_H_55_FeN_6_O_14_; exact mass: 863.71 (calculated)].

#### 4.2.4. Conjugation of Tetrazine-PEG_5_ Motif

Iron protected FSC (1.0 mg, 1.28 µmol), MAFC (2.0 mg, 2.43 µmol) or DAFC (2.0 mg, 2.32 µmol) were dissolved in 500 µL anhydrous DMF and after addition of Tetrazine-PEG_5_-NHS ester, 1.5 equivalents (2.10 mg, 3.48 µmol) in case of DAFC, 2.5 equivalents (3.67 mg, 6.08 µmol) in case of MAFC and 3.5 equivalents (2.71 mg, 4.48 µmol) in case of FSC, pH was adjusted to 9.0 using DIPEA and the reaction mixtures were maintained for 4 h at RT. Finally the organic solvent was evaporated and the crude mixture was used without further purification.

#### 4.2.5. Demetallation

For the purpose of iron removal, corresponding conjugates were dissolved in 1 mL H_2_O/ACN solvent 50% (*v*/*v*) and 1 mL of aqueous Na_2_EDTA solution (200 mM) was added. The resulting mixtures were stirred for 4 h at ambient temperature followed by preparative RP-HPLC purification to give slightly to intensively pink coloured, iron free FSC-based Tz-conjugates after lyophilisation.

- DAFC-PEG_5_-Tz (=Tz-monomer): 2.35 mg [1.81 µmol, 78%], gradient B (*t*_R_ = 31.1 min); Analytical data: RP-HPLC *t*_R_ = 11.5 min; MALDI TOF-MS: *m*/*z* [M + H]^+^ = 1303.95 [C_60_H_89_N_11_O_21_; exact mass: 1300.41 (calculated)]
- MAFC-(PEG_5_-Tz)_2_ (=Tz-dimer): 3.65 mg [2.09 µmol, 86%], gradient B (*t*_R_ = 35.3 min); Analytical data: RP-HPLC *t*_R_ = 12.5 min; MALDI TOF-MS: *m*/*z* [M + H]^+^ = 1748.25 [C_81_H_118_N_16_O_27_; exact mass: 1747.89 (calculated)]
- FSC-(PEG_5_-Tz)_3_ (=Tz-trimer): 1.68 mg [0.77 µmol, 60%], gradient B (*t*_R_ = 37.9 min); Analytical data: RP-HPLC *t*_R_ = 13.2 min; MALDI TOF-MS: *m*/*z* [M + H]^+^ = 2200.20 [C_102_H_147_N_21_O_33_; exact mass: 2195.38 (calculated)]

#### 4.2.6. Modification of Rituximab (RTX)

Rituximab was obtained in solution (Mabthera^®^, 10 mg/mL, Roche Pharma AG, Grenzach-Wyhlen, Germany) and a PD-10 (GE Healthcare, Vienna, Austria) size exclusion column was used for buffer exchange according to manufacturer’s protocol to give RTX in 0.1 M NaHCO_3_ solution (7 mg/mL). For the conjugation of *trans*-cyclooctene (TCO), 2 mL of the RTX solution were mixed with 20 molar equivalent of TCO-NHS ester dissolved in DMSO and the reaction was stirred for 30 min at ambient temperature followed by incubation overnight at 4 °C under light exclusion. Subsequently, the modified antibody (RTX-TCO) was purified using size exclusion chromatography (PD-10) to give 10 mg of RTX-TCO dissolved in PBS. The antibody was treated following the same procedure as described above without adding TCO-NHS ester, in order to obtain a non-modified RTX counterpart as negative control.

### 4.3. Fluorescence Activated Cell-Sorting (FACS)

The humanoid lymphoblast-like CD20-expressing B-lymphocyte cells (Raji cells) were purchased from American Type Culture Collection (ATCC, Manassas, VA, USA). Flow cytometric analysis of CD20 expression was evaluated on (Raji cells) adjusted to 5 × 10^5^ cells/sample in DMEM (including 10% FCS, 1% Pen/Strep). Cells were incubated with FC-Block (containing CD16/CD32 (1:200), e-Bioscience, Thermo Fisher Scientific, Vienna, Austria) for 15 min at 4 °C in order to avoid signals from non-specific binding. The following antibodies were used: RTX-TCO and RTX, both at 20 µM (f. c.). As secondary antibody a APC-labelled anti-human IgG Fc (clone: HP6017; BioLedgend, San Diego, CA, USA) at a dilution of 1:100 was used. Antibodies were incubated for at least 15 min at 4 °C. FACS analysis was performed on BD LSRFortessa™ (Cell Analyzer, BD Bioscience, San Jose, CA, USA). As internal control samples unstained or stained with either primary or secondary antibodies were analyzed. FACS-data was analyzed by FlowJo v10 software.

### 4.4. Radiolabelling of FSC-Based Tz-Conjugates with Gallium-68

Gallium-68 was obtained as [^68^Ga]GaCl_3_ (gallium chloride) by fractioned elution of a ^68^Ge/^68^Ga-generator (IGG100, nominal activity 1100 MBq, Eckert & Ziegler, Berlin, Germany) with 0.1 M hydrochloric acid (HCl, Rotem Industries Ltd., Beer-Sheva, Israel). Hereafter, 500 µL of eluate (100 MBq) were mixed with 100 µL sodium acetate solution (1.14 M) to give a pH of 4.5 followed by addition of 10 µg (4.55–7.68 nmol) of corresponding FSC-Tz conjugate. After 5 min incubation at RT the radiolabelling solution was analyzed using radio-RP-HPLC.

### 4.5. In Vitro Characterization.

#### 4.5.1. Distribution Coefficient (LogD)

To determine the distribution of the ^68^Ga-labelled conjugates between an organic (octanol) and aqueous (PBS) layer, aliquots (50 µL) of the tracers (~5 µM) were diluted in 1 mL of octanol/PBS (1:1, *v*/*v*). The mixture was vortexed at 1400 rpm (MS 3 basic vortexer, IKA, Staufen, Germany) for 15 min at RT followed by centrifugation for 2 min at 4500 rpm. Subsequently, aliquots (50 µL) of both layers were collected and measured in the gamma counter (Wizard2 3”, Perkin Elmer, Waltham, MA, USA) followed by logD calculation (*n* = 3, six replicates).

#### 4.5.2. Protein Binding

Serum protein binding was determined using Sephadex G-50 (GE Healthcare Vienna, Austria) size exclusion chromatography [38]. Aliquots (50 µL, *n* = 3) of the radioligand solution (~10 µM) were incubated in 450 µL freshly prepared human serum or 450 µL PBS (controls) and were kept at 37 °C. After 1, 2, and 4 h aliquots (25 µL) were directly transferred to the column (MicroSpin G-50, GE Healthcare) and after centrifugation (2 min, 2000 rcf) the column containing the free conjugate and the eluate containing the protein-bound conjugate were measured in the gamma counter. The percent of activity in both fractions was calculated thereafter.

#### 4.5.3. Stability Studies in Human Serum

Stability of the radioligands was evaluated in human serum as described in [38]. Briefly, 50 µL of the radioligand solution (~10 µM, *n* = 2) were mixed with 950 µL freshly prepared serum or 950 µL PBS (controls). The mixtures were then maintained at 37 °C. At designated time points, 1, 2 and 4 h respectively, aliquots (100 µL) were mixed with 0.1% TFA/ACN, centrifuged for 2 min at 14 × 10^3^ rcf. The supernatant was diluted with H_2_O (1:1, *v*/*v*) and analyzed by analytical RP-HPLC for decomposition without filtration prior to injection.

#### 4.5.4. Competitive Binding Assay

Binding on immobilized RTX-TCO was conducted using high protein-binding capacity Nunc MaxiSorp™ 96-well plates (Thermo Fisher Scientific, Vienna, Austria). Coating was performed by adding 20 µg of antibody (RTX or RTX-TCO) dissolved in 100 µL coating buffer (0.1 M NaHCO_3_, pH 8.5) to each well and after 2 h incubation at RT the plate was left at 4 °C overnight both under the exclusion of light. After removal of the coating solution 200 µL of blocking buffer (1% BSA in PBS) was added, left for 1 h at room temperature and subsequently each well was washed twice with 200 µL binding buffer (0.1% BSA in PBS). Hereafter, the radioligand was mixed with increasing concentrations of the competitor (=non-labelled conjugate), diluted in binding buffer and 100 µL of the mixture was added to each well. After 30 min at RT the supernatant was removed, each well was washed three times with 150 µL binding buffer and the coating film was finally detached with 2 × 150 µL of hot (80 °C) 2 N sodium hydroxide (NaOH). The NaOH fraction was taken for gamma counter measurement to determine the percentage of binding in contrast to the standard followed by non-liner curve fitting using Origin 6.1 software (Origin Inc., Northampton, MA, USA) to calculate the apparent half maximum inhibitory concentration of the competitor (*n* = 3, 4 replicates).

#### 4.5.5. Cell Binding

Raji-cells were seeded in tissue culture flasks (Cellstar; Greiner Bio-One, Kremsmuenster, Austria) using RPMI-1640 medium supplemented with fetal bovine serum (FBS) to a final concentration of 10% (*v*/*v*). In order to do studies on cell binding 10 × 10^6^ cells were washed twice with fresh media, diluted with PBS to a final concentration of 1 × 10^6^ cells per mL and 500 µL of cell suspension was transferred to Eppendorf tubes. Hereafter, 50 µL of RTX-TCO or non-modified RTX as negative control (both 0.5 µM) were added and the cell suspension was maintained at 37 °C under gentle shaking. After 1 h the suspension was centrifuged (2 min, 11 × 10^3^ rcf), the supernatant was discarded, the cells were washed twice and finally resuspended with 450 µL PBS. Subsequently, 50 µL of the radioligand solution (22 nM in PBS) was added and the suspension was incubated for 30 min at 37 °C. After centrifugation and two washing steps with 600 µL PBS, the cells were resuspended in 500 µL PBS and transferred to polypropylene vials for gamma counter measurement followed by calculation from cell-associated activity in comparison to the standard (*n* = 3, six replicates).

### 4.6. In Vivo Characterization

#### 4.6.1. Ethics Statement

All animal experiments were performed in accordance with regulations and guidelines of the Austrian animal protection laws and the Czech Animal Protection Act (No. 246/1992), with approval of the Austrian Ministry of Science (BMWF-66.011/0161-WF/V/3b/2016), the Czech Ministry of Education, Youth, and Sports (MSMT-18724/2016-2), and the institutional Animal Welfare Committee of the Faculty of Medicine and Dentistry of Palacky University in Olomouc.

#### 4.6.2. Biodistribution Studies

Biodistribution of ^68^Ga-labelled conjugates was conducted in healthy 5-week-old female BALB/c mice (Charles River Laboratories, Sulzfeld, Germany). Animals (*n* = 3) were injected via lateral tail vain with 1 nmol of conjugate and a total activity of approximately 6 MBq. Mice were sacrificed by cervical dislocation 1 h p.i. followed by collection of the main organs and tissue, subsequent gamma counter measurement and calculation of the percentage of injected activity per gram tissue (% IA/g).

#### 4.6.3. Imaging Studies

MicroPET/CT images were acquired with an Albira PET/SPECT/CT small animal imaging system (Bruker Biospin Corporation, Woodbridge, CT, USA). Mice were pre-treated by intramuscular (i.m.) injection of 50 µL of RTX-TCO to the left hind muscle and 50 µL of RTX to the right hind muscle. Pre-treated mice were retro-orbitally (r.o.) injected with radiolabelled tracer in a dose of 5–10 MBq corresponding to 1–2 μg of conjugate per animal 5 h after the pre-treatment. Anaesthetized (2% isoflurane (FORANE, Abbott Laboratories, Abbott Park, IL, USA)) animals were placed in a prone position in the Albira system before the start of imaging. Static PET/CT images were acquired over 30 min starting 90 min p.i. A 10-min PET scan (axial FOV 148 mm) was performed, followed by a double CT scan (axial FOV 65 mm, 45 kVp, 400 μA, at 400 projections). Scans were reconstructed with the Albira software (Bruker Biospin Corporation) using the maximum likelihood expectation maximization (MLEM) and filtered backprojection (FBP) algorithms. After reconstruction, acquired data was viewed and analyzed with PMOD software (PMOD Technologies Ltd., Zurich, Switzerland). 3D volume rendered images were obtained using VolView software (Kitware, Clifton Park, NY, USA).

## Figures and Tables

**Table 1:** ^1^H-NMR data (chemical shifts and integrals) of characteristic signals of FSC-based Tz-conjugates and *N*,*N*′,*N*′′-triacetylfusarinine (TAFC) as a reference.

	FSC Subunit		Acetyl	PEG_5_-Tz Subunit				
	3× CH	3× CH_3_	CH_3_	Tetrazine	p-Phenylen		NH-CH_2_	NH-CH_2_
	6.3 ppm	1.86 ppm	1.83 ppm	10.56 ppm	8.44 ppm	7.53 ppm	8.50 ppm	4.40 ppm
Tz-monomer	3 H	9 H	6 H	1 H	2 H	2 H	1 H	2 H
Tz-dimer	3 H	9 H	3 H	2 H	4 H	4 H	2 H	4 H
Tz-trimer	3 H	9 H	none	3 H	6 H	6 H	3 H	6 H
TAFC	3 H	9 H	9 H	none	none	none	none	none

**Table 2:** Distribution coefficient (logD) and protein binding of ^68^Ga-labelled FSC-based Tz-conjugates.

^68^Ga-Labelled Compound	LogD (pH 7.4)	Protein Binding (%)		
		1 h	2 h	4 h
Tz-monomer	−1.64 ± 0.02	61.8 ± 0.2	63.8 ± 2.1	64.0 ± 1.4
Tz-dimer	−1.35 ± 0.01	67.0 ± 2.4	65.9 ± 1.3	68.4 ± 0.3
Tz-trimer	−1.00 ± 0.06	70.5 ± 0.7	69.5 ± 0.4	67.8 ± 0.4

Data are presented as mean ± SD (*n* = 3)

## Supplementary Materials

The following are available online at http://www.mdpi.com/1424-8247/11/4/102/s1, Figure S1: ^1^H-NMR spectrum of DAFC-PEG_5_-Tz (Tz-monomer), Figure S2: ^1^H-NMR spectrum of of MAFC-(PEG_5_-Tz)_2_ (Tz-dimer), Figure S3: ^1^H-NMR spectrum of FSC-(PEG_5_-Tz)_3_ (Tz-trimer), Figure S4: Representative RP-HPLC chromatograms of mono- and multimeric FSC-Tz conjugates (A, UV/vis chromatograms) and their ^68^Ga-labelled counterparts (B, radio-chromatograms), Figure S5: Representative radio-RP-HPLC chromatograms of the stability assessment of ^68^Ga-labelled mono- and multimeric FSC-Tz conjugates incubated with fresh human serum (A) and PBS (B), Figure S6: Fluorescence-activated cell sorting of unstained Raji cells, RTX, RTX-TCO, secondary antibody (APC), RTX + APC and RTX-TCO + APC (order from left to right).

## Abbreviations

ACN	acetonitrile
BSA	bovine serum albumin
CD	cluster of differentiation
DAFC	*N*,*N*′-diacetylfusarinine C
DIPEA	*N,N*-diisopropylamine
DMF	*N,N*-dimethylformamide
EDTA	ethylenediaminetetraacetic acid
FSC	fusarinine C
IEDDA	inverse electron-demand Diels-Alder
i.m.	intramuscular
mAb	monoclonal antibody
MAFC	*N*-monoacetylfusarinine C
NHS	*N*-hydroxysuccinimide
p.i.	post injection
PBS	phosphate buffered saline
PET/CT	positron emission computed tomography
r.o.	retro-orbitally
RP-HPLC	reversed phase high performance liquid chromatography
RT	room temperature
RTX	rituximab
TCO	*trans*-cyclooctene
TFA	trifluoracetic acid
Tz	tetrazine
