# Supplementary figures and images for: Pretargeted Imaging with Gallium-68—Improving the Binding Capability by Increasing the Number of Tetrazine Motifs
Source: Pharmaceuticals (Basel). 2018 Oct 11;11(4):102. doi: 10.3390/ph11040102 (PMC6316846; doi:10.3390/ph11040102)

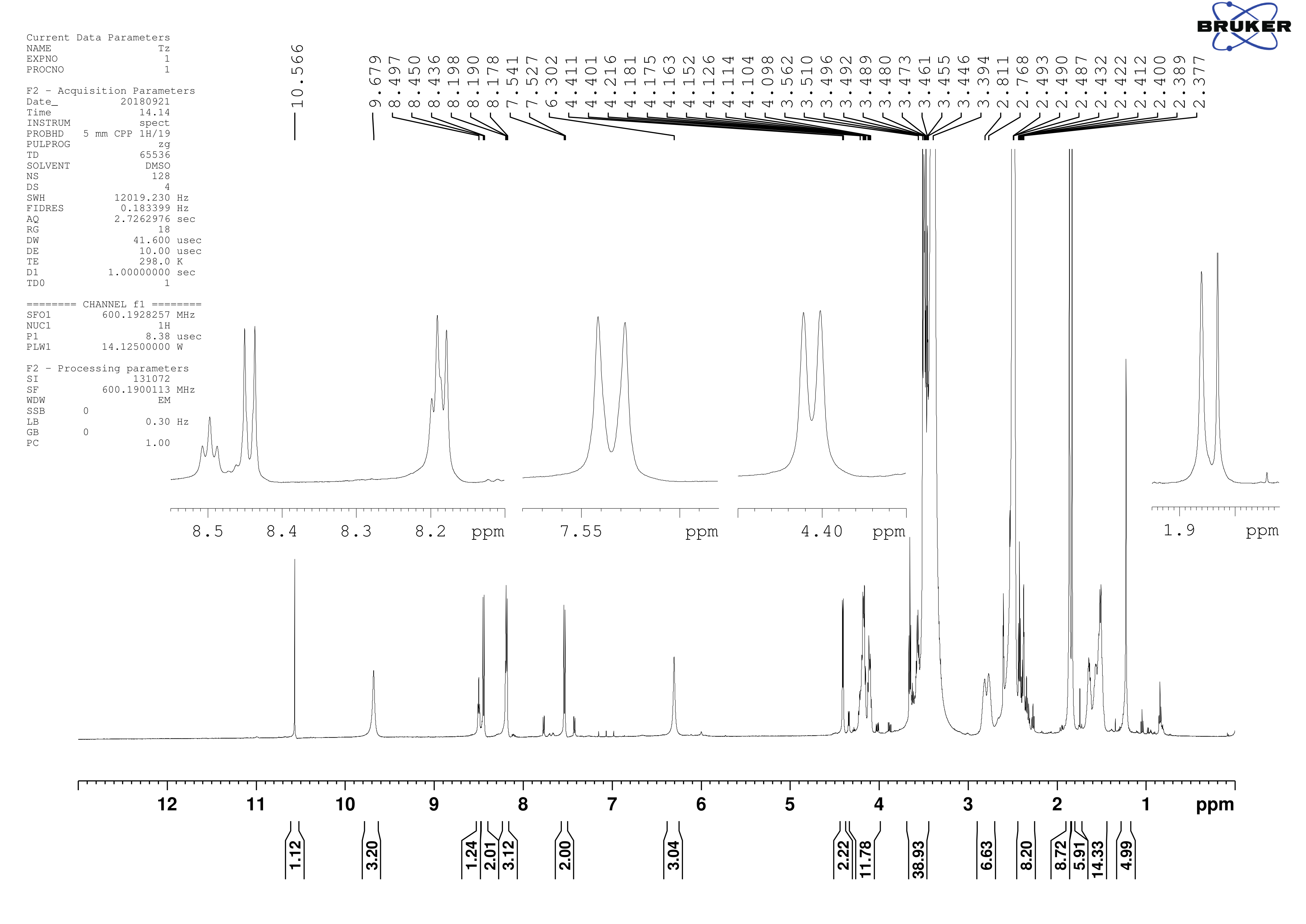

Supplement: Supplementary file 1 [file pharmaceuticals-11-00102-s001.zip › Figure S1.tif]

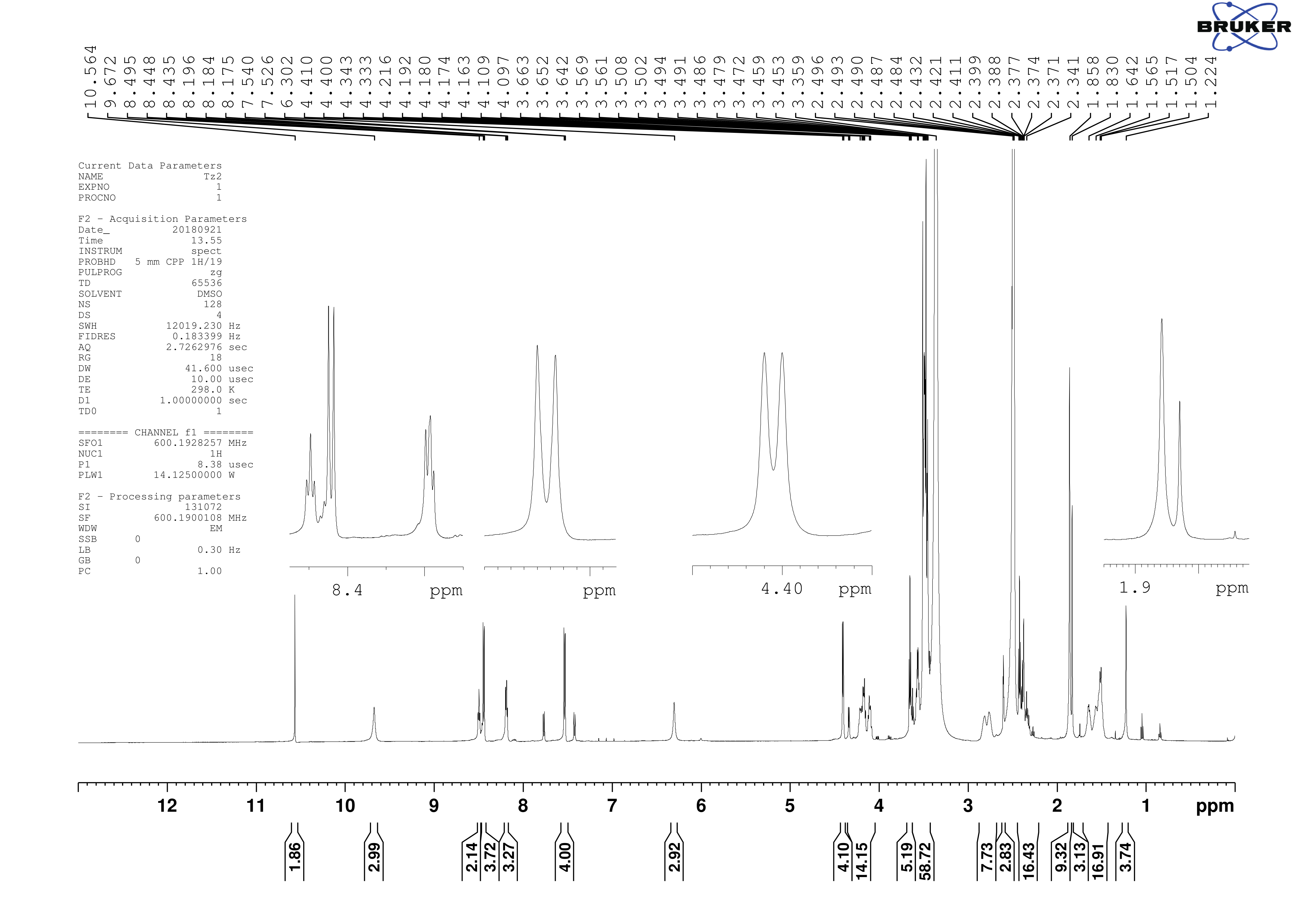

Supplement: Supplementary file 1 [file pharmaceuticals-11-00102-s001.zip › Figure S2.tif]

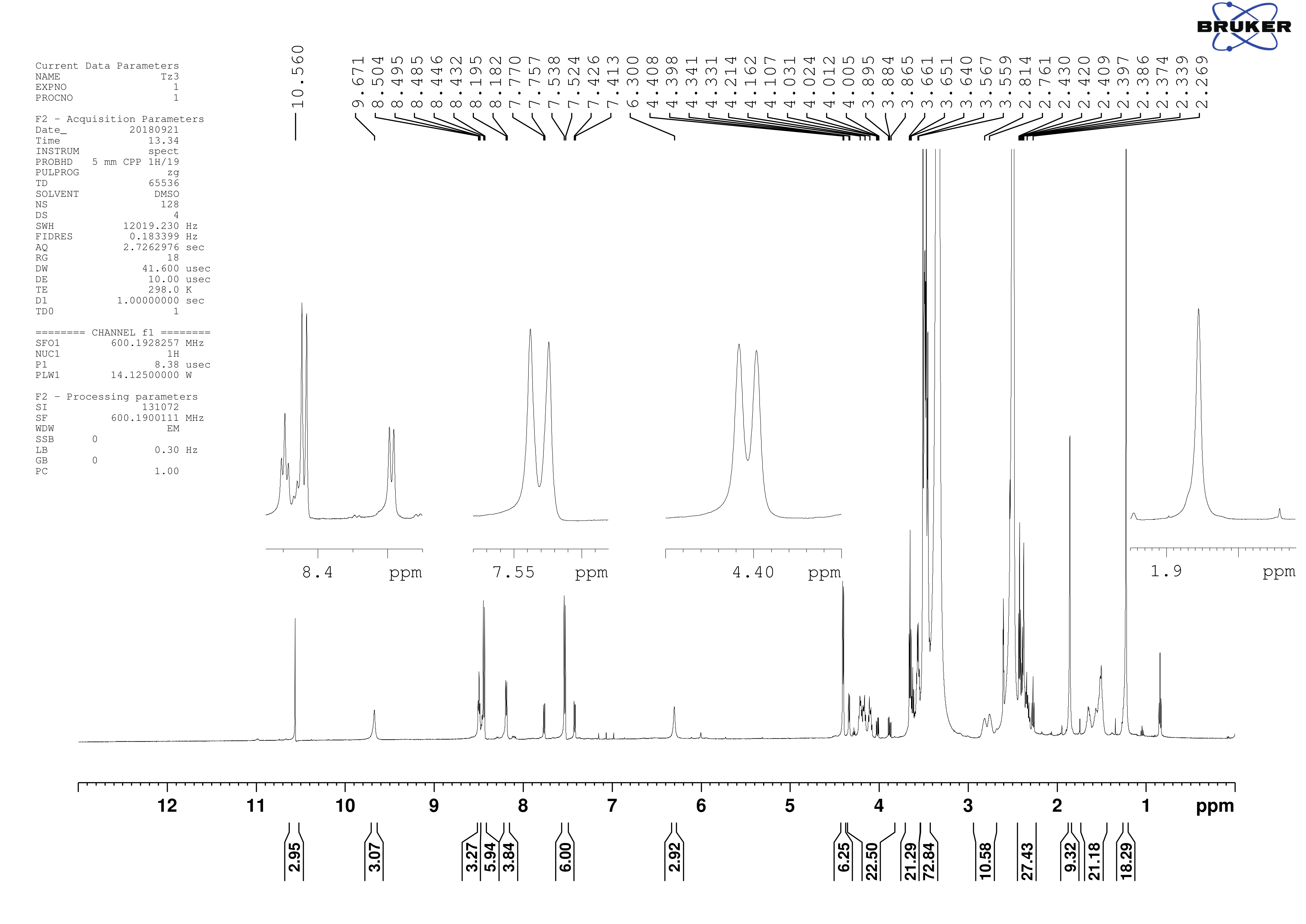

Supplement: Supplementary file 1 [file pharmaceuticals-11-00102-s001.zip › Figure S3.tif]

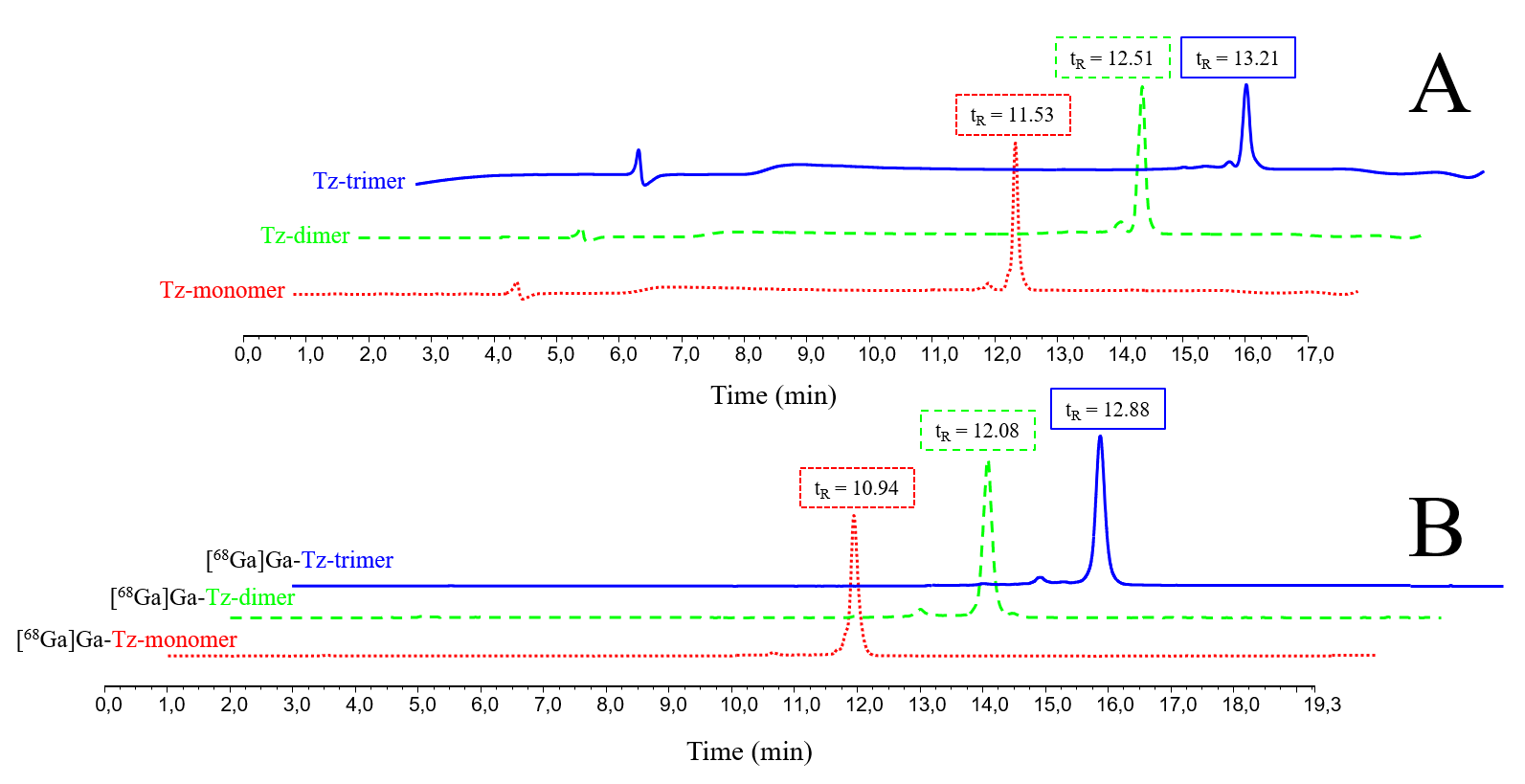

Supplement: Supplementary file 1 [file pharmaceuticals-11-00102-s001.zip › Figure S4.tif]

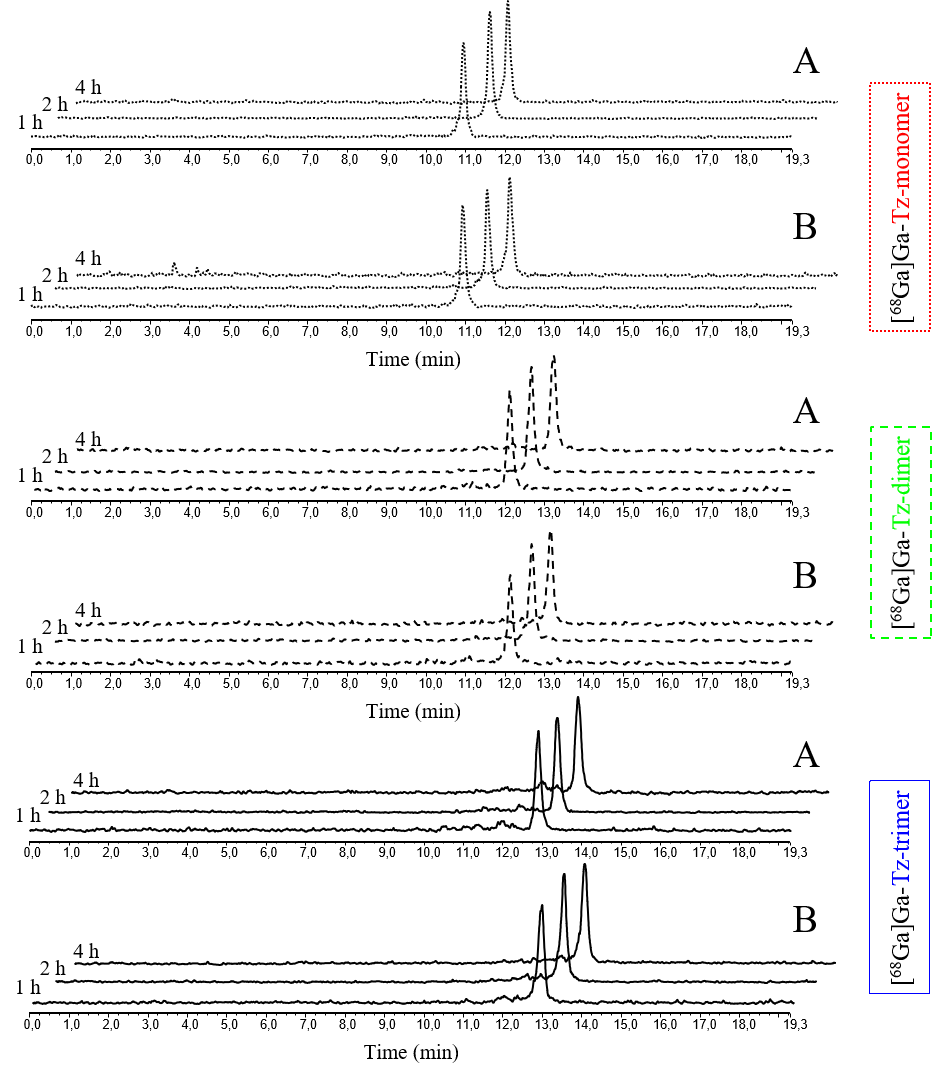

Supplement: Supplementary file 1 [file pharmaceuticals-11-00102-s001.zip › Figure S5.tif]

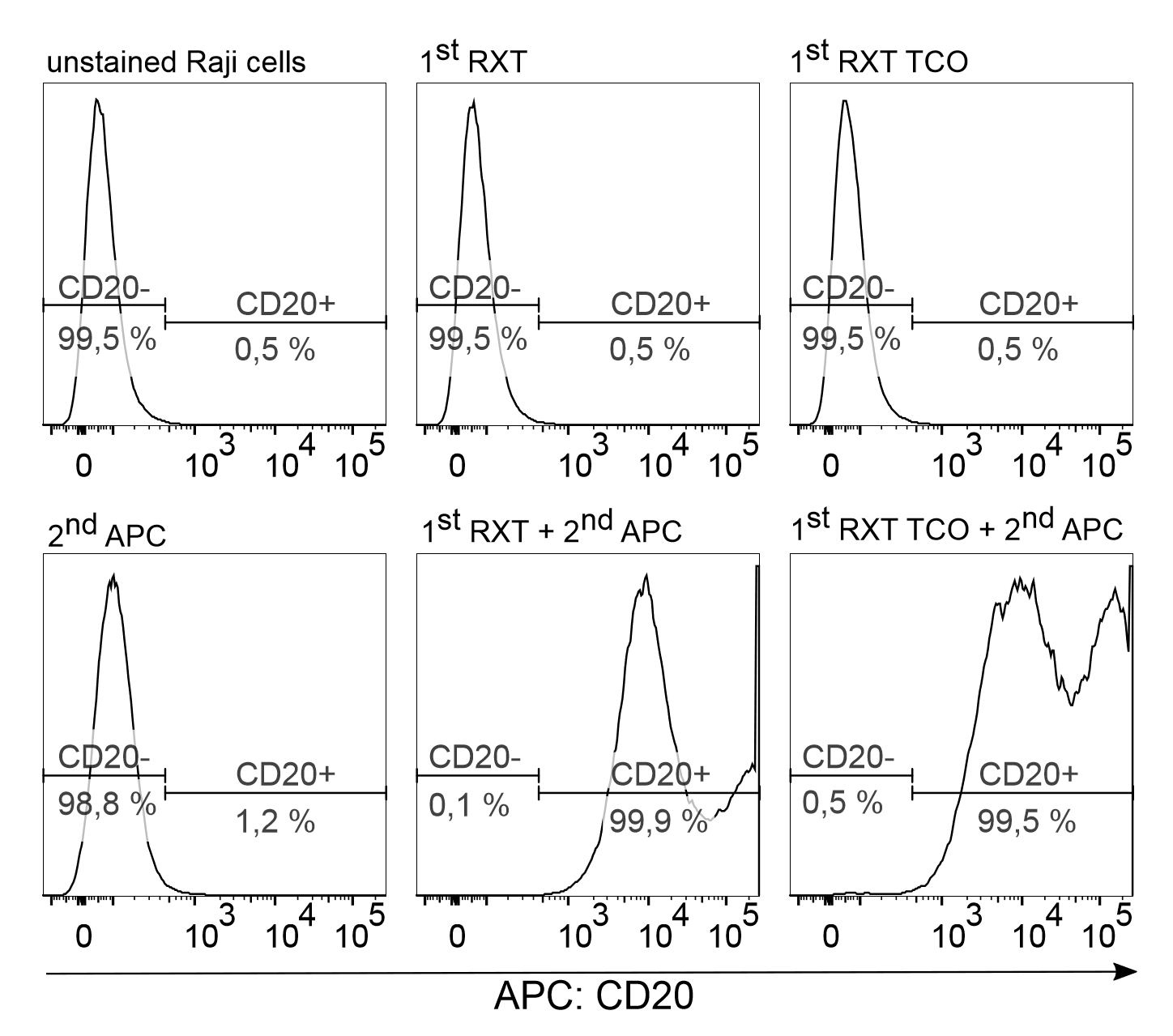

Supplement: Supplementary file 1 [file pharmaceuticals-11-00102-s001.zip › Figure S6.png]

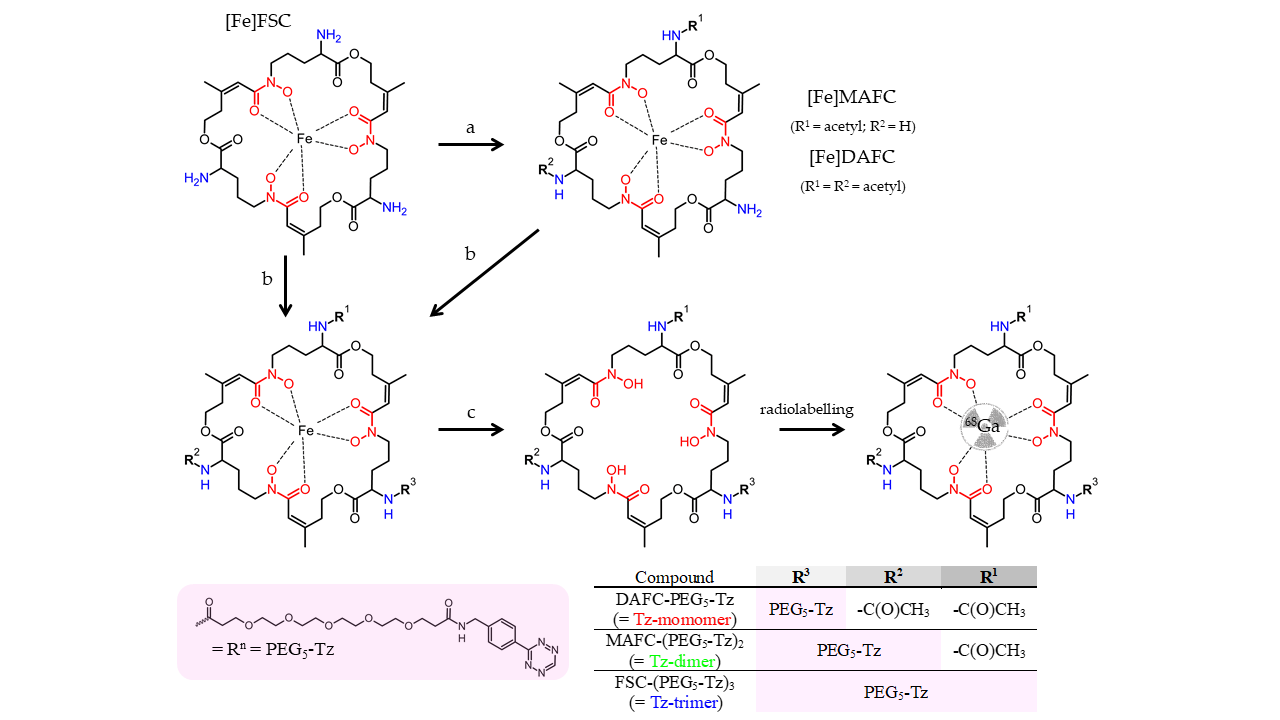

Supplement: Supplementary file 1 [file pharmaceuticals-11-00102-s001.zip › Scheme I.tif]
